# Preventive Effect of Dexamethasone Therapy on the Transient Hypoparathyroidism through Total Thyroidectomy 

**Published:** 2019-03

**Authors:** Mohsen Kolahdouzan, Bijan Iraj, Mohammad Eslamian, Mohammad Harandizadeh, Rokhsareh Meamar

**Affiliations:** 1 *Isfahan Endocrine and Metabolism Research Center, Isfahan University of Medical Sciences, Isfahan, Iran.*; 2 *Isfahan Clinical Toxicology Research Center, Isfahan University of Medical Sciences, Isfahan, Iran.*

**Keywords:** Drug Utilization, Hypoparathyroidism, surgery, dexamethasone

## Abstract

**Introduction:**

This study aimed to investigate whether pre-operative dexamethasone could ameliorate transient hypoparathyroidism outcome through total thyroidectomy.

**Materials and Methods:**

This randomized clinical trial study was conducted on 128 patients underwent total thyroidectomy from March 2014 to April 2015. Patients were randomly assigned to two groups of experimental receiving 8 mg IV of intravenous dexamethasone (n=45) 45 min before skin incision treatment and control (n=83). After the surgery, all patients were evaluated for clinical and laboratory hypocalcaemia.

**Results:**

Post-operative transient biochemical hypoparathyroidism and hypocalcaemia did not occur more often in the control group, compared to the dexamethasone group while controlling for the baseline variables. However, there was a significant difference in phosphorus level between the dexamethasone and control groups (P=0.028). A total of 50 (39.1%) patients developed hypocalcaemia after surgery. Moreover, post-operative symptomatic hypocalcemia occurred more frequently in the control group (68%) compared to the dexamethasone group (32%); however, this difference was not statistically significant (P=0.54).

**Conclusion:**

The pre-operative administration of dexamethasone reduced post-operative hypocalcemia rate. It is essential to conduct future studies with validated means for better results.

## Introduction

Total thyroidectomy is currently an accepted surgical method for benign and malignant thyroidal disorders (1-3). 

However, it might enhance the risk of post-operative complications following an extensive resection (4). The complication rates of thyroidectomy for transient hypoparathyroidism are reported in a wide range of 6.9-46% (5-7), which leads to post-operative hypocalcemia with the incidence of 1.6-50% (8-9). Hypocalcaemia is the most prevalent and sometimes the most intensive complication observed after total thyroidectomy (10). The primary cause of hypocalcaemia is secondary to hypoparathyroidism due to devascularization, the accidental removal of one or more parathyroid glands during surgery, or a damage to parathyroid glands as a result of lymphadenectomy (10,11). 

Therefore, an appropriate surgical technique during total thyroidectomy could easily prevent surgery complications, particularly related to parathyroid glands (12). On the other hand, post-operative transient hypocalcaemia generally responds favorably to calcium and vitamin D supplementation within a few days or one week leading to prolonged hospitalization duration. When prophylactic consumption is administered in patients with normal calcium levels, it carries the risk of triggering hypercalcemia (11), which explains the need for alternative therapy.Several clinical trials in various main and minor surgical procedures have indicated that pretreatment with single-dose glucocorticoid modifies potentially important biological effects of perioperative inflammatory responses. This issue declines the post-surgical inflammatory response and attenuates complications and length of hospitalization (13,14). 

However, other effects of glucocorticoids, including analgesic, immune-modulating, and antiemetic properties, are well-known (15). Dexamethasone is introduced as safe and effective corticosteroid medication with a suggested single perioperative dose for reduction of post-operative nausea, vomiting, and pain (16-18). 

Recently, a double-blinded randomized trial revealed that the administration of dexamethasone could decrease the incidence and severity of a post-operative sore throat during swallowing after thyroidectomy (19). However, there are few surveys in the literature about the application of dexamethasone in post - operative hypoparathyroidism. There was only one study, which showed that pretreatment with single-dose dexamethasone could influence post-operative transient hypoparathyroidism (16). Therefore, the present study aimed to investigate whether pre-operative dexamethasone could ameliorate transient hypoparathyroidism outcome through total thyroidectomy.

## Materials and Methods

**Study Design:**

This randomized clinical trial study was conducted from March 2014 to April 2015 at the surgery ward in Alzahra Hospital, Isfahan, Iran (IRCT2017011329726N3). A total of 128 patients were admitted to the hospital, and consequently underwent total thyroidectomy. Almost most cases had thyroid tumor but approximately 10% (n=15) of patients were selected for thyroidectomy due to other causes, such as multinodular goiter and hyperthyroidism resistant to treatment. Patients with a history of the parathyroid disease, renal failure, and unilateral thyroid lobectomy were excluded from the study. All operations were carried out by an experienced surgeon in the high-volume center for thyroid surgery. The study protocol was sanctioned by the Ethics Committee of the Faculty of Medicine of the University of Medical Sciences, Isfahan, Iran, and all patients filled out a written informed consent in order to participate in the study. This consent form was designed according to the Declaration of Helsinki.Demographic data, including age, gender, histological diagnosis, and pathology results (data regarding the observation of any parathyroid tissues in the specimen) were collected from all patients. Total thyroidectomy was performed by a capsular dissection technique. The indication for surgery was determined by an endocrine subspecialty internist and an experienced surgeon. The determination for surgery was based on clinical findings and thyroid surgery reference guidelines. During this prospective study, subjects were randomized and divided into two groups of treatment (n=45) and control (n=83). The individuals who attended the two groups were matched for age and gender.

Patients were randomized to obtain intravenous dexamethasone, 8mg IV (Decadron; Merck Sharp & Dohme) 45 min before skin incision (16,20). All patients received general anesthesia similarly. The average operation time was approximately 75 min. In the post-operative anesthesia care unit (PACU), vital signs (i.e., blood pressure, pulse, respiration, pulse oximetry, and adequate answering) were screened every 15 min by an experienced specialist. Patients were discharged from the PACU when vital signs were regulated and stable. After the surgery, all patients were evaluated for clinical and laboratory hypocalcaemia. Patients with any symptoms of hypocalcaemia were not discharged on the first day after the surgery, and oral supplement therapy was initiated for them. All patients without any symptoms of hypocalcaemia were discharged on the first post-operative day. Furthermore, patients were asked to contact the researchers and begin the consumption of the oral calcium supplements and vitamin D analog (calcitriol, Rocaltrol) when they have any symptoms and signs of hypocalcaemia. As a result, if there had been no improvement of the symptoms of hypocalcaemia with about 8 tablets of calcium carbonate, subjects were re-admitted to the hospital for intravenous replacement therapy.

Instances of symptomatic hypocalcemia were defined as patients developing any of the symptoms, such as circumoral numbness, tingling of the fingers and toes, paresthesia or muscle irritability or a positive Chvostek sign. Replacement therapy with oral calcium supplements and vitamin D analog (calcitriol, Rocaltrol) began in patients with clinical hypocalcemia. Laboratory hypocalcemia was described as at least 1 serum calcium determination below 8.1 mg/dL (reference range of 8.1-10.4 mg/dL). If patients progressed to symptomatic hypocalcemia or when the serum calcium level was less than 8.1 mg/dL, it was better to administrate oral calcium supplementation with or without vitamin D analog. 

An intravenous calcium gluconate 10% infusion was prescribed for significant hypocalcemic symptoms or until oral therapy was ineffective. Patients were discharged when the serum calcium level was higher than 8.1 mg/dL.

*Analysis of Laboratory Concentrations*

 Furthermore, laboratory data, such as serum calcium, serum phosphorus, vitamin D level and serum parathyroid hormone (PTH) levels before surgery, post-operative calcium, and PTH levels, were measured at 1, 6, and 24 h of operation. Patients were followed-up daily from the day before the operation until the end of the first post-operative week. The serum levels of calcium (normal range of 8.2–10.6mg/dL), phosphorus (normal range of 2.5–4.5mg/dL) were performed by spectrophotometric methods (Hitachi 902 autoanalyzer). Other laboratory data of peripheral blood, including 25OHD (vitamin D is defined as a deficiency in this study as the 25OHD concentration of less than 20.0 ng/mL) and PTH (normal range: 10– 65 IU/L), were analyzed by enzyme immunoassay (Biomerica, CA, and IDS, UK). 

**Statistical analysis**

Quantitative data were reported as mean or median (range) and categorical data as frequency (percentage). The normality of quantitative data was evaluated using Kolmogorov–Smirnov test and Q-Q plot. Nonnormal data were subjected to logarithmic transformation. 

Between-group comparisons based on quantitative data were conducted using independent t-test or analysis of covariance (ANCOVA) (adjustment was made for baseline values) and for categorical data using the Chi-square or Fisher exact test. Within-group comparisons were employed using paired samples t-test. All the statistical calculations were carried out with SPSS software (version 15, SPSS Inc., Chicago, IL, USA).

## Results

A total of 128 patients were available for the analysis. There were 45 patients in the dexamethasone group and 83 were in the control group (without receiving any treatment). 

Table 1 shows the demographic characteristics of patients in the dexamethasone and control groups. The only significant variations between groups were pre-operative PTH level (P<0.05).

**Table 1:** Characteristics of the investigated patients in the dexamethasone and control groups

	**Control group**	**Dexamethasone group**	**P-value**
Age	Mean±SD	Mean±SD	0.45
	45.87±12.57	43.55±12.01	
Gender	N (F/M)	N (F/M)	0.47
	83 (70/13)	45 (40/5)	
Ca (mg/dL) pre-operative	Mean±SD	Mean±SD	0.83
	9.25±0.56	9.2±0.56	
	Median [min-max]	Median [min-max]	
	9.2 [8.1-11]	9.3 [8.1-10.9]	
Phosphor(mg/dL) pre-operative	Mean±SD	Mean±SD	0.82
	3.53±0.51	3.5±0.67	
	Median [min-max]	Median [min-max]	
	3.55 [2.6-4.7]	3.6 [2.3-4.8]	
PTH pre-operative	Mean±SD	Mean±SD	0.004
	58.2±26.31	38.59±22.55	
	Median [min-max]	Median [min-max]	
	51 [13.8-138.1]	31 [11.7-82]	

PTH: parathyroid hormone Ca: Calcium

The comparison of calcium and PTH levels in pre-operative and post-operative status revealed a significant difference between the two groups (P<0.001; (Table.2).

 However, after adjusting for the baseline values, we did not observe any significant difference between the two groups. There was a significant difference in phosphorus level between the dexamethasone group and control groups (P=0.028). The PTH level in the pre-operative state had a significant difference between the control group (58.2±26.31) and the dexamethasone group (38.59±22.55; P=0.004) but after adjusting for baseline values there was no significant difference between the two groups (P=0.0601).

**Table 2:** Pre- and post-operative calcium, phosphor, parathyroid hormone levels between dexamethasone and control groups

		**Pre-operative**	**Post-operative**	**P-value** *	**P-value ** **
Control group	Calcium (mg/dL)	Mean ±SD	Mean ±SD	<0.001	0.337
		9.25±0.56	8.67±0.33		
		Median [min-max]	Median [min-max]		
		9.2 [8.1-11]	8.6 [7.8-10]		
Dexamethasone group		Mean ±SD	Mean ±SD	<0.001	
		9.2±0.56	8.6±0.78		
		Median [min-max]	Median [min-max]		
		9.3 [8.1-10.9]	8.55 [6.8-10.2]		
P-value***		0.83	0.47		
Control group	Phosphor (mg/dL)	Mean ±SD	Mean ±SD	0.53	0.028
		3.53±0.51	3.5±0.49		
		Median [min-max]	Median [min-max]		
		3.55 [2.6-4.7]	3.6 [2.7-4.5]		
Dexamethasone group		Mean ±SD	Mean ±SD	0.097	
		3.5±0.67	3.9±0.8		
		Median [min-max]	Median [min-max]		
		3.6 [2.3-4.8]	3.9 [2.5-5.8]		
P-value		0.82	0.005		
Control group	PTH	Mean ±SD	Mean ±SD	<0.001	0.601
		58.2±26.31	19.7±16.47		
		Median [min-max]	Median [min-max]		
		51 [13.8-138.1]	16.5 [0.1-62.65]		
Dexamethasone group		Mean ±SD	Mean ±SD	<0.001	
		38.59±22.55	18.41±14.45		
		Median [min-max]	Median [min-max]		
		31 [11.7-82]	16.2 [2.1-80]		
P-value		0.004	0.66		

* Resulted from paired t-test

** Resulted from analysis of covariance (ANCOVA) adjusting for baseline values

*** Resulted from independent t-test PTH: parathyroid hormone

**Fig 1:**
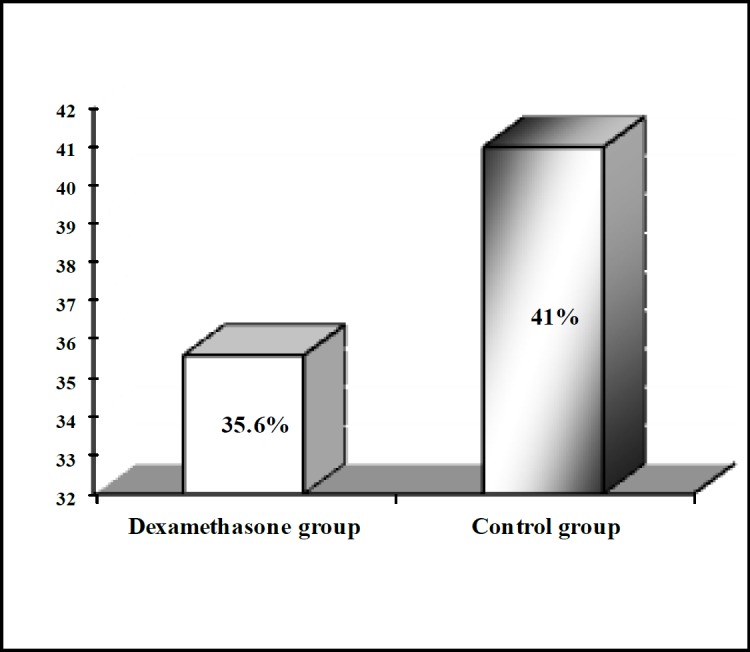
Frequency distribution of symptomatic hypocalcemia between dexamethasone and control groups

A total of 50 patients (39.1%) developed hypocalcaemia after surgery. Post-operative symptomatic hypocalcemia occurred more often in the control group (68%), compared to the dexamethasone group (32%); however, this difference was not statistically significant (P=0.54); (Fig.1).

In addition, subgroup analysis was performed in dexamethasone treatment based on the existence or lack of symptomatic hypocalcemia. In patients with or without symptomatic hypocalcaemia, a significant difference was observed in calcium and PTH levels between pre- and post-operative levels (Table.3).

It was found that declining in only post-operative serum calcium level in patients who developed symptomatic hypocalcaemia more frequently happened than the patients without symptomatic hypocalcaemia with (P=0.05) or without adjusting for baseline calcium level (P=0.02; Table.3). 

**Table 3:** Pre- and post-operative calcium, phosphor, parathyroid hormone levels between patients with and without symptomatic hypocalcemia in the dexamethasone group

		**Pre-operative**	**Post-operative**	**P-value** *	**P-value ** **
Patients with symptomatic hypocalcemia n=9	Calcium (mg/dL)	Mean ±SD	Mean ±SD	0.001	0.05
		9.2±0.78	8.2±0.78		
		Median [min-max]	Median [min-max]		
		9 [8.2-10.9]	8.5 [6.8-9.5]		
Patients Without symptomatic hypocalcemia n=29		Mean ±SD	Mean ±SD	0.01	
		9.3±0.42	8.8±0.72		
		Median [min-max]	Median [min-max]		
		9.4 [8.1-10]	8.7 [7.5-10.2]		
P-value***		0.72	0.02		
Patients with symptomatic hypocalcemia n=8	Phosphor (mg/dL)	Mean ±SD	Mean ±SD	0.7	0.73
		3.8±0.59	4.02±0.91		
		Median [min-max]	Median [min-max]		
		3.8 [2.8-4.8]	3.9 [2.7-5.8]		
Patients Without symptomatic hypocalcemia n=29		Mean ±SD	Mean ±SD	0.096	
		3.3±0.66	3.8±0.75		
		Median [min-max]	Median [min-max]		
		3.5 [2.3-4.6]	4 [2.5-5]		
P-value		0.09	0.53		
Patients with symptomatic hypocalcemia n=16	PTH	Mean ±SD	Mean ±SD	<0.0001	0.186
		49.66±18.18	14.58±9.88		
		Median [min-max]	Median [min-max]		
		58.2 [11.7-71]	12.77 [2.1-31.35]		
Patients Without symptomatic hypocalcemia n=29		Mean ±SD	Mean ±SD	<0.0001	
		50.73±16.72	20.53±16.21		
		Median [min-max]	Median [min-max]		
		58.2 [14-82]	17.6 [3-80]		
**P-**value		0.84	0.189		

* Resulted from paired t-test

** Resulted from analysis of covariance (ANCOVA) adjusting for baseline values

*** Resulted from independent T-test

## Discussion

In the present study, post-operative transient biochemical hypoparathyroidism and hypocalcaemia did not occur more often in the control group, compared to the dexamethasone group. However, a non-significant lower occurrence rate in post-operative symptomatic hypocalcemia was observed in the dexamethasone group when compared with the control group.

A major and occasionally severe complication after total thyroidectomy is the clinically manifested post-operative hypoparathyroidism (9,10,21,22). The mechanism of transient hypoparathyroidism is not completely clear yet, but the most accepted explanation is related to the manipulation of the parathyroid glands resulted from the partial disrupt of the blood supply of the glands, leading to transient hypocalcaemia (10,12).

It is obvious that the sensitive predictor of post-operative hypocalcaemia is a significant reduction in post-operative blood calcium. The early diagnosis and prophylaxis treatment of hypocalcaemia is the avoidance of symptomatic hypocalcaemia and a significant reduction of hospital stay and hospitalization costs (11).There are inadequate data for deterministic conclusions to be drawn regarding the fact that the effectiveness of prophylactic treatment with the drug could reduce post-operative transient hypocalcaemia.

However, in one study, it was revealed that prophylactic treatment with single-dose dexamethasone (8mg IV) could influence post-operative transient hypoparathyroidism (16). To the best knowledge of the researchers, the present study was the first study indicating the efficacy of dexamethasone for the prevention of transient hypoparathyroidism in patients undergoing only total thyroidectomy. The chance of traumatic edema or vasospasm, which might have induced edema and/or temporary hypoparathyroidism, could increase during surgery. Based on experimental and clinical data, steroid administration may prevent or reduce edema due to the surgical trauma (16,23). 

In other words, glucocorticoids act as physiologic modifiers of the post-operative inflammatory response by reducing both vascular reactivities to vasoconstrictors and the induced cellular immune response mainly regulated by cytokine functions(15,24). 

Cytokines play a major role in immunological function following the surgery (24). In the present study, it seemed that dexamethasone reduced the induced immunologic transient response to surgery in parathyroid gland causing no significant reduction of transient hypocalcaemia rate in the dexamethasone group. The obtained results of the current study were consistent with a study conducted by Nasiri et al. indicating that prophylactic treatment with dexamethasone reduced vocal dysfunction only on the first day after thyroidectomy (25). It can be concluded that dexamethasone transiently could influence thyroid surgical outcomes. 

Moreover, we measured calcium and PTH several times both in the pre-operative and post-operative stage. The comparison of calcium and PTH levels in pre-operative and post-operative status revealed a significant difference between the control and dexamethasone groups without adjusting for baseline values. In patients who developed hypoparathyroidism, PTH levels decreased more rapidly when compared with the decline in serum calcium level (26). Four hours after total thyroidectomy, there was a significant change of PTH level, which guide physicians as the valuable predictor of hypocalcemia for treatment of high-risk patients in order to decline the risk of post-operative hypocalcemia development and earlier hospital discharge (27). The relationship between pre-operative serum calcium levels and post-operative hypocalcaemia is unclear (28).

A recent meta-analysis presented there was no specific correlation between pre-operative serum calcium levels and post-operative hypocalcaemia (29). On one hand, post-operative hypocalcemia had a crucial role for diagnosis and appropriate management of hypocalcemia. Finally, it seemed quite logical for the pre- and post-operative determination of calcium and PTH for a suitable treatment of hypocalcemia.

On the other hand, it is essential to consider the time of administration. One meta-analysis in 2015 presented that the effectiveness of a single dose of pre-operative dexamethasone and time of administration are still unknown (30). However, in most of the studies, this drug was administered 20-45 min before the induction of anesthesia (30). According to the fact that glucocorticoids bind to the intracellular receptors and depend on the route of administration, the onset of biological activity takes usually one to two hours (15,31). Consistent with these data, for the full post-operative benefit of the treatment, we administered dexamethasone 45 min before surgery.

In conclusion, according to previous studies, based on the effectiveness and safety of dexamethasone (8 mg IV) for pain, fatigue, vomiting, and vocal dysfunction, we evaluated transient hypoparathyroidism in patients undergoing total thyroidectomy (17,20,30,32). 

A non-significant post-operative symptomatic hypocalcemia was less observed in the dexamethasone group, compared to the control group. It seems that precise care during surgery is more important than dexamethasone administration. 

## Conclusion

 The pre-operative administration of dexamethasone reduced post-operative hypocalcemia rate. It is suggested to further the study with validated means for better results. 
